# Yokukansan, a Traditional Japanese Medicine, Adjusts Glutamate Signaling in Cultured Keratinocytes

**DOI:** 10.1155/2014/364092

**Published:** 2014-09-17

**Authors:** Maki Wakabayashi, Toshio Hasegawa, Takuji Yamaguchi, Naoko Funakushi, Hajime Suto, Rie Ueki, Hiroyuki Kobayashi, Hideoki Ogawa, Shigaku Ikeda

**Affiliations:** ^1^Department of Dermatology and Allergology, Juntendo University Graduate School of Medicine, 2-1-1 Hongo, Bunkyo-ku, Tokyo 113-8421, Japan; ^2^Center for Advanced Kampo Medicine and Clinical Research, Juntendo University Graduate School of Medicine, 2-1-1 Hongo, Bunkyo-ku, Tokyo 113-8421, Japan; ^3^Tsumura Research Laboratories, Tsumura & Co, 3586 Yoshiwara, Ami-machi, Inashiki-gun, Ibaraki 300-0075, Japan; ^4^Atopy (Allergy) Research Center, Juntendo University Graduate School of Medicine, 2-1-1 Hongo, Bunkyo-ku, Tokyo 113-8421, Japan

## Abstract

Glutamate plays an important role in skin barrier signaling. In our previous study, Yokukansan (YKS) affected glutamate receptors in NC/Nga mice and was ameliorated in atopic dermatitis lesions. The aim of this study was to assess the effect of YKS on skin and cultured human keratinocytes. Glutamate concentrations in skin of YKS-treated and nontreated NC/Nga mice were measured. Then, glutamate release from cultured keratinocytes was measured, and extracellular glutamate concentrations in YKS-stimulated cultured human keratinocytes were determined. The mRNA expression levels of NMDA receptor 2D (NMDAR2D) and glutamate aspartate transporter (GLAST) were also determined in YKS-stimulated cultured keratinocytes. The glutamate concentrations and dermatitis scores increased in conventional mice, whereas they decreased in YKS-treated mice. Glutamate concentrations in cell supernatants of cultured keratinocytes increased proportionally to the cell density. However, they decreased dose-dependently with YKS. YKS stimulation increased NMDAR2D in a concentration-dependent manner. Conversely, GLAST decreased in response to YKS. Our findings indicate that YKS affects peripheral glutamate signaling in keratinocytes. Glutamine is essential as a transmitter, and dermatitis lesions might produce and release excess glutamate. This study suggests that, in keratinocytes, YKS controls extracellular glutamate concentrations, suppresses N-methyl-D-aspartate (NMDA) receptors, and activates glutamate transport.

## 1. Introduction

Atopic dermatitis (AD) is associated with skin barrier dysfunction, and acute disruption of the skin barrier results in epidermal DNA synthesis and cytokine production. Glutamate is known to play an important role in skin barrier signaling during barrier disruption [1]. Glutamate signaling is predominantly associated with excitatory synaptic neurotransmission in the central nervous system (CNS) [2]. In the central nervous system, glutamate is produced by glia and Schwann cells [3] or released at the synaptic gap by excitatory neurons; at this location, it binds to the various subtypes of glutamate receptors [4]. Thus, neurotransmission is thought to occur by signaling through glutamate in the central nervous system. In contrast to the nervous system, the origin of the transmitter glutamate within the epidermis has not yet been elucidated. However, accumulating data suggest that glutamate might also act as a cell-signaling molecule in nonneuronal tissues, such as the skin. Indeed, there is evidence of a glutamate-mediated signaling pathway in the epidermis [5–7].

Yokukansan (YKS) is a traditional Japanese medicine that is collectively known as Kampo. It is composed of 7 types of dried medical herbs. YKS has been approved by the Ministry of Health, Labor, and Welfare of Japan as a remedy for neurosis, insomnia, and irritability in children. According to recent reports, YKS improved the behavioral and psychological symptoms of dementia, such as hallucinations, agitation, and aggressiveness, in patients with Alzheimer's disease, dementia with Lewy bodies, and other forms of senile dementia. It has recently been shown that YKS regulates glutamate signaling in the central nervous system [8, 9], and it is a novel NMDA receptor antagonist (i.e., a glutamate receptor antagonist) [10]. Although it has been previously assumed that glutamate receptors exist predominantly in the nervous system, growing evidence in recent years has suggested that they also exist in nonneuronal cells, such as keratinocytes and melanocytes [1, 5, 11–17]. Furthermore, in nonneuronal tissue, YKS reportedly exerts antiallergic effects on IgE-mediated triphasic cutaneous (immediate phase response, late phase response, and very late phase response) reactions in socially isolated mice.

We have previously demonstrated that YKS inhibited scratching behavior and ameliorates atopic dermatitis- (AD-) like lesions in NC/Nga mice [18], which have been regarded as an excellent AD model because they develop AD-like skin lesions spontaneously under conventional conditions but not under specific pathogen-free (SPF) conditions [19]. In addition, YKS suppresses the activity of NMDA receptors and decreases transepidermal water loss (TEWL) in the skin of NC/Nga mice [18]. However, the mechanism of the effectiveness of YKS is still unclear. From these results, we hypothesized that one of the mechanisms underlying the effect of YKS might be related to glutamate function, which could lead to improved dermatitis. Functional glutamate signaling requires the expression of glutamate receptors (e.g., NMDA receptor 2D (NMDAR2D)) and transporters (e.g., glutamate transporter 1 (GLT-1) and glutamate aspartate transporter (GLAST)). YKS might affect glutamate, glutamate receptors, and glutamate transporters.

Thus, the aim of this study was to evaluate the effects of YKS on the glutamate signaling pathway in the skin of NC/Nga mice and cultured human keratinocytes.

## 2. Materials and Methods

### 2.1. Animals

Male NC/Nga mice (10 weeks of age) were purchased from Japan SLC Inc. (Shizuoka, Japan) and were maintained under conventional conditions (conventional control and YKS-treated groups) and under SPF conditions (SPF control group). The mice were individually housed (under social isolation stress) throughout the experiment. The animal room was maintained at 24 ± 2°C, 55 ± 10% relative humidity, and a 12-12 h light-dark cycle. All mice were allowed access to water and food ad libitum. Individual cages measured 9(*w*) × 13(*h*) × 20(*d*) cm in size. All procedures performed on animals were approved by the Institutional Animal Care and Use Committee of Juntendo University under approval number 220014.

### 2.2. Cell Culture

Normal human epidermal keratinocytes purchased from Kurabo Industries (Osaka, Japan) were cultured in serum-free keratinocyte growth medium, HuMedia-KG2 (Kurabo Industries) containing human epidermal growth factor (0.1 ng/mL), insulin (10 mg/mL), hydrocortisone (0.5 mg/mL), gentamycin (50 mg/mL), amphotericin B (50 ng/mL), and bovine brain pituitary extract (0.4% vol/vol). After the cells had been serially passaged at 60–70% confluence, experiments were conducted using subconfluent cells at passage 3 or 4 in the proliferative phase at 60–80% confluence. After medium removal, the cells were washed twice with phosphate-buffered saline (PBS) before culture for 24 hours in HuMedia supplemented only with antibiotics. Keratinocytes were subsequently stimulated with YKS.

### 2.3. YKS and Reagents

**Figure 1:**
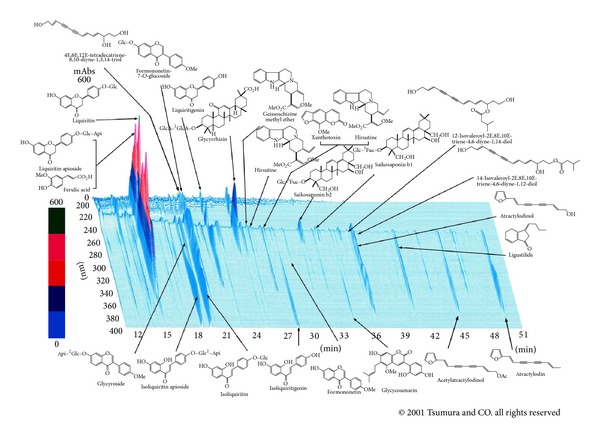
Chemical profile of Yokukansan. Chemical profile of Yokukansan analysed by three-dimensional high performance liquid chromatography (HPLC). Each peak of Yokukansan in the HPLC profile was identified by comparison of the retention times and UV spectra of chemically defined standard compounds.

YKS (in general, patients take 2.5 g of YKS powder 3 times daily) contained 7 dried medicinal herbs:* Atractylodes lancea* rhizome (4.0 g rhizome of* Atractylodes lancea* De Candolle),* Poria* sclerotium (4.0 g sclerotium of* Poria cocos* Wolf),* Cnidium* rhizome (3.0 g rhizome of* Cnidium officinale* Makino), Japanese* Angelica* root (3.0 g root of* Angelica acutiloba* Kitagawa),* Bupleurum* root (2.0 g root of* Bupleurum falcatum* Linné),* Glycyrrhiza* root (1.5 g root and stolon of* Glycyrrhiza uralensis* Fisher), and* Uncaria* thorn (3.0 g thorn of* Uncaria rhynchophylla* Miquel) (Figure 1). The dry-powdered extracts of YKS were supplied by Tsumura & Co (Tokyo, Japan).

The reagents, including glutamate, used in the cell culture experiments were purchased from Sigma-Aldrich (St. Louis, MO, USA). Other chemicals were purchased from commercial sources.

We confirmed that the concentrations of glutamate and YKS used in this study did not significantly affect cell viability (data not shown), employing a cell counting kit-8 from Dojindo (Tokyo, Japan).

### 2.4. Evaluation of the Severity of Dermatitis and Examination of YKS Effects on Glutamate Production in the Skin of NC/Nga Mice

First, the investigations were performed using NC/Nga mice. The mice were divided into 3 groups: specific pathogen-free (SPF) control, conventional control, and YKS- (Tsumura & Co, Tokyo, Japan) treated (*n* = 5 per group). The SPF control mice were isolated under SPF conditions. The conventional control mice were maintained under conventional conditions without YKS treatment. The seven medicinal herbs were extracted with purified water at 95°C for 1 h, and the extraction solution was separated from the insoluble waste and was concentrated by removing water under reduced pressure. The spray-drying technique was used to produce a dried extract powder. The mice in the YKS-treated group were given water containing YKS at the dosage of 1% (1.7 g/kg) for 6 weeks under conventional conditions. The control mice were given drug-free water ad libitum. We only used NC/Nga mice with mild skin lesions for these experiments, excluding those with either less severe or more severe lesions.

The severity of skin lesions was examined and scored. Skin lesions on the dorsal skin were assessed according to the following 4 symptoms: erythema, edema, erosion, and dryness. The sum was considered to be the individual score (0, no symptom; 1, mild; 2, moderate; 3, severe).

To measure glutamate, dorsal skin specimens were harvested with a surgical knife, homogenized, and subjected to cell lysis in 1 mL PBS. The supernatant was extracted by centrifugation (4°C, 10,000 RPM, 30 min). The total levels of secreted and intracellularly stored glutamate were examined using a glutamate assay kit (Abcam, Cambridge, United Kingdom). The glutamate concentrations were determined based on the protein content per unit (*μ*M/*μ*g protein) using a BCA kit (Thermo Scientific, Yokohama, Japan).

### 2.5. Measurement of Glutamate Release from Cultured Keratinocytes

Second, the investigations were performed using cultured keratinocytes. To determine the glutamate concentrations in the culture supernatant, the keratinocytes were cultured in 35 mm culture dishes. When the cultures reached certain stages of confluence, the cell supernatant was removed. The following measurement points were defined: nonconfluent (approximately 30% of the area covered with cells), subconfluent (approximately 60–80% of the area covered with cells), and confluent (90–100% of the area covered by cells). Glutamate concentrations were measured using the glutamate assay kit (Abcam, Cambridge, United Kingdom).

### 2.6. Examination of YKS Effects on Glutamate Production and Release in Cultured Keratinocytes

Next, keratinocytes were cultured with various concentrations of YKS (125, 250, and 500 *μ*g/mL), which had been filtered through a 0.20 *μ*m filter, or medium alone in 6-well tissue culture plates. At 90% confluence, the glutamate released into the cell-free supernatants from nonstimulated cultures or those stimulated with 125–500 *μ*g/mL YKS was measured using Abcam glutamate assay kits.

### 2.7. Examination of YKS Effects on NMDAR, GLAST, and GLT-1 mRNA Expressions in Cultured Keratinocytes

Finally, keratinocytes with 125, 250, and 500 *μ*g/mL of YKS or medium alone were cultured in 6-well plates, according to the procedure described in the previous section. NMDA receptor 2D (NMDAR2D), glutamate transporter 1 (GLT-1), and glutamate aspartate transporter (GLAST) mRNA levels were determined via real-time polymerase chain reaction (RT-PCR) when the cells were confluent.

Total RNA was extracted from the keratinocytes using an RNeasy Plus Micro Kit (Qiagen, Hilden, Germany), and first-strand cDNA was synthesized from 1 *μ*g of total RNA using ReverTra Ace qPCR RT Kit (Toyobo, Osaka, Japan). RT-PCR was performed using the TaqMan Universal PCR Master Mix (Applied Biosystems, Branchburg, NJ, USA). Amplification and detection of the mRNA were performed using a StepOne Plus RT-PCR System (Biosystems). All RT-PCR reactions were performed 6 times each, and the gene expression changes were reported as fold increases relative to the untreated controls.

### 2.8. Statistical Analysis

The statistical analysis was performed with an analysis of variance (ANOVA) followed by the appropriate post hoc test or with Student's *t*-test. The statistical analyses were performed with GraphPad Prism for Windows (Prism 5, GraphPad Software, San Diego, CA, USA). A value of *P* < 0.05 was considered to indicate a statistically significant difference. The results are presented as the means ± SD.

## 3. Results

### 3.1. Assessment of Dermatitis Score and Glutamate Concentrations in the Skin of NC/Nga Mice

**Figure 2:**
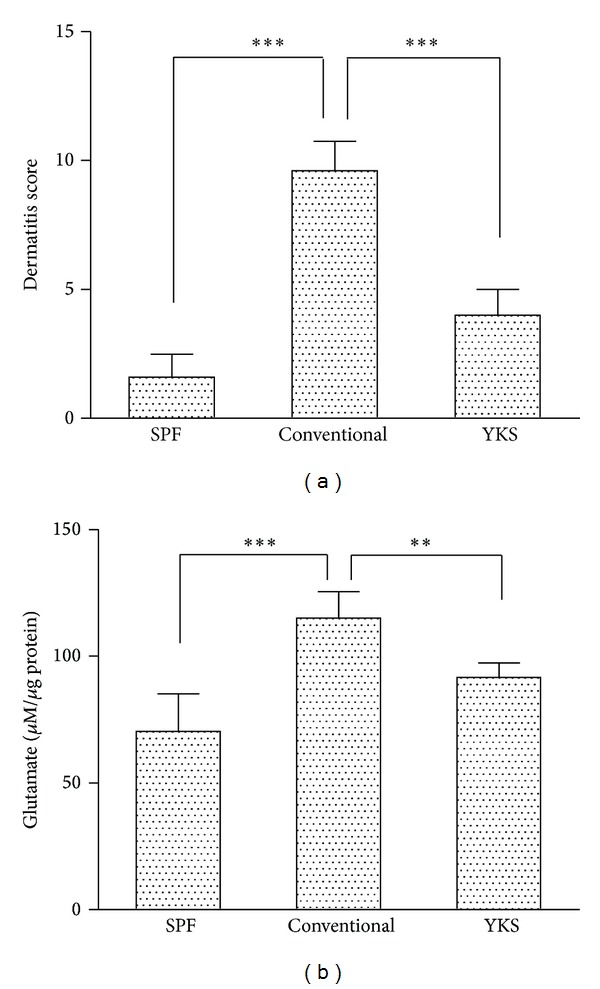
AD-like lesions produce and release glutamate in the skin of NC/Nga mice. The dermatitis scores of the conventional control mice were aggravated. YKS-treated mice significantly inhibited the aggravation of skin lesions in NC/Nga mice. (a) The glutamate concentrations in the skin of the NC/Nga mice, as determined by ELISA, were significantly increased in the conventional control mice compared with the SPF control mice. However, the glutamate concentrations were significantly decreased in the YKS-treated mice compared with the conventional control mice. (b) Measurements were compared between the SPF control and conventional control groups (conventional control: nontreated, YKS-treated). *P < 0.05, **P < 0.01, and ***P < 0.001.

The dermatitis scores of the conventional control mice were aggravated. YKS significantly inhibited the aggravation of skin lesions in the NC/Nga mice. The dermatitis score of the SPF control mice was not severe (Figure 2(a)).

The glutamate concentrations in the skin of NC/Nga mice were significantly increased in the conventional control mice compared with the SPF control mice. However, glutamate concentrations were significantly decreased in the YKS-treated mice compared with the conventional control mice (Figure 2(b)).

### 3.2. Time Course of Glutamate Concentrations in Cultured Keratinocytes

**Figure 3:**
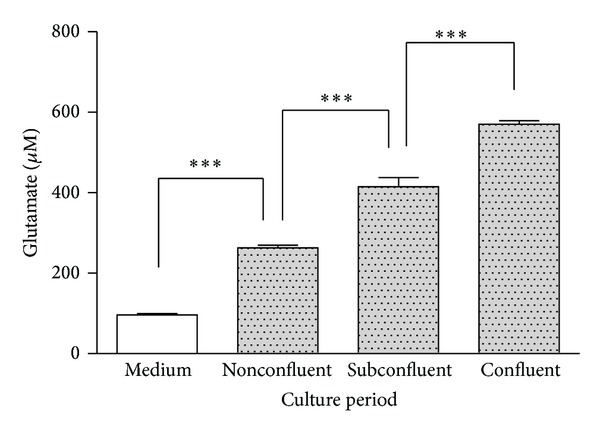
Keratinocytes are able to produce and release glutamate. Cultured keratinocytes were observed at different stages of confluence. When the culture reached a certain stage of confluence, the glutamate concentrations were measured by ELISA. The following measurement points were defined: nonconfluent (approximately 30% of the area covered by cells), subconfluent (approximately 70% of the area covered with cells), and confluent (90–100% of the area covered by cells). The extracellular glutamate concentration increased as the cell density increased. The measurements were compared between cell-free conditions and cells that had reached certain stages of confluence (*n* = 6). *P < 0.05, **P < 0.01, and ***P < 0.001.

The extracellular concentration of glutamate increased as the cell density increased (Figure 3). We speculate that this increase originates from an outflow of glutamate from the keratinocytes.

### 3.3. Effect of YKS on Glutamate Concentrations in Cultured Keratinocytes

**Figure 4:**
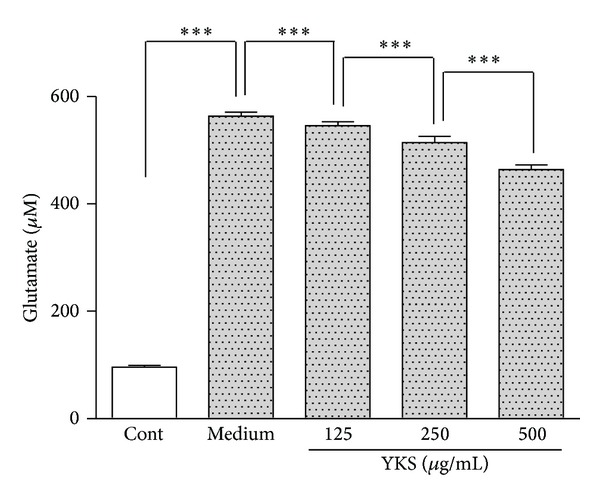
YKS decrease excessive glutamate production and release in cultured keratinocytes. Keratinocytes were cultured until the cells became confluent in normal medium or media containing various concentrations of YKS (125–500 *μ*g/mL). At 90% confluence, the concentrations of glutamate in the culture supernatants were determined by ELISA. The glutamate concentrations decreased in the keratinocytes stimulated with YKS. The measurements were compared between medium (YKS 0 *μ*g/mL) and YKS 125–500 *μ*g/mL (*n* = 6). Cont: control (medium alone without cells) *P < 0.05, **P < 0.01, and ***P < 0.001.

The glutamate concentrations in the medium decreased for the keratinocytes treated with YKS (125, 250, and 500 *μ*g/mL) (Figure 4). These results suggested that YKS reduced excessive glutamate production by cultured keratinocytes.

### 3.4. YKS Regulates the Glutamate Receptor NMDAR2D and the Glutamate Transporter GLAST mRNA Expression in Cultured Keratinocytes

**Figure 5:**
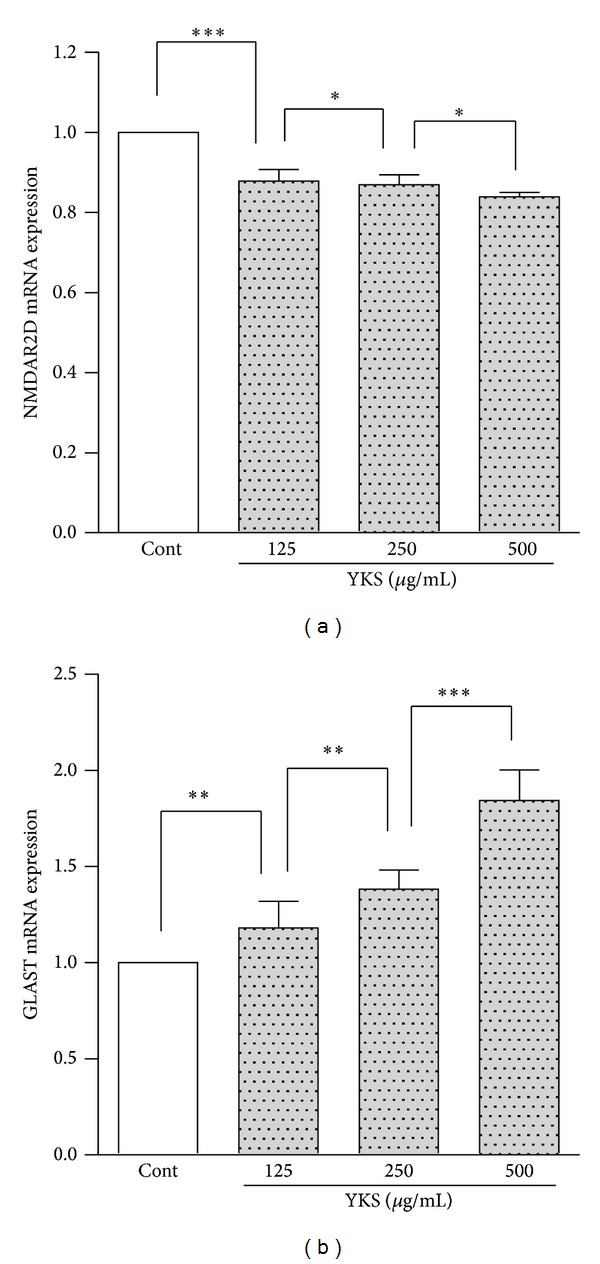
YKS regulates NMDAR2D and GLAST mRNA expression in cultured keratinocytes. Human keratinocytes were incubated with 125–500 *μ*g/mL of YKS or medium alone until the cells became confluent. Following incubation, total RNA was extracted and reverse transcribed into cDNA and then RT-PCR was performed. The NMDAR2D (a) and GLAST (b) mRNA expression levels were measured using RT-PCR. NMDAR2D mRNA expression decreased in response to YKS stimulation (125–500 *μ*g/mL) in a concentration-dependent manner. GLAST mRNA levels increased following the addition of YKS in a concentration-dependent manner. The measurements were compared between the stimulated and nonstimulated cells (Cont; control). (*n* = 6) *P < 0.05, **P < 0.01, and ***P < 0.001.

NMDAR2D mRNA expression in the cultured keratinocytes decreased in response to YKS stimulation (125–500 *μ*g/mL) in a concentration-dependent manner (Figure 5(a)). In contrast, the GLAST mRNA level in the cultured keratinocytes increased after adding YKS in a concentration-dependent manner (Figure 5(b)). The GLT-1 mRNA was not expressed (data not shown).

## 4. Discussion

We previously demonstrated that YKS ameliorates AD-like lesions in NC/Nga mice [18, 20]. In addition, YKS suppressed the activity of NMDA receptors and decreased the numbers of mast cells infiltrating the skin of NC/Nga mice [18].

In this study, we observed increased glutamate concentrations in the AD-like lesion of conventional control mice. However, we observed that the glutamate concentrations were decreased in the skin of YKS-treated mice. The glutamate concentration and dermatitis score were correlated. Dermatitis lesions can produce and release excess glutamate. These results suggested that YKS reduced the excessive glutamate production in NC/Nga mouse skin.

In the cultured keratinocytes, the extracellular concentrations of glutamate increased as the cell density increased. We speculate that this increase originated from an outflow of glutamate from the keratinocytes.

Furthermore, the YKS-treated keratinocytes also showed decreased glutamate concentrations in the cell supernatant. HaCaT cells have also been shown to produce and release glutamate, which can act as a transmitter on epidermal glutamate receptors [14]. Because both autocrine and paracrine mechanisms are possible in keratinocytes [21], keratinocytes might stimulate their own glutamate receptors through glutamate release.

A recent study demonstrated that YKS was a novel NMDA receptor antagonist [10] and had antiallergic properties. Among several components in YKS, isoliquiritigenin was reported to be an NMDA receptor antagonist [10]. Because the NMDA receptor is important for glutamate signaling, we hypothesized that YKS would inhibit the function of NMDA receptors by adjusting the extracellular glutamate concentration.

NMDA receptors, which belong to the family of ionotropic glutamate receptors, appear to be uniformly distributed over the surfaces of basal keratinocytes [5]. They consist of subunits that are designated as NMDAR1, NMDAR2, and NMDAR3. In addition, there are 4 known variants of NMDAR2 designated as NMDAR2 A–D [22, 23]. Fischer et al. demonstrated that NMDA1 and NMDAR2D are likely key components of the NMDA receptors in human keratinocytes [17]. According to their report, mRNA expressions of NMDAR2A, NMDAR2B, and NMDAR2C were not detected in either keratinocytes or HaCaT cells. The authors showed that NMDAR2D was specifically expressed in cultured keratinocytes [17]. Thus, we examined NMDAR2D (NMDAR1 was nearly undetectable). In this study, we demonstrated that YKS suppresses the activity of NMDAR2D-type receptors in cultured keratinocytes. In future experiments, we would like to demonstrate whether YKS can suppress the expression of NMDAR1 and NMDAR2D in the mouse model. Further study is necessary to investigate NMDAR2D-specific knockdown experiment. NMDA receptors reside on keratinocytes and are known to be involved in the process of skin barrier repair [1, 5, 16, 24]. MK-801, a known antagonist of NMDA receptors, has been suggested to reduce the autocrine effect of glutamate and, consequently, to block delayed barrier recovery [1]. Thus, YKS, which is also an NMDA antagonist, might improve skin barrier function.

Functional glutamate signaling also requires the expression of glutamate transporters to remove and recycle released glutamate, thereby inactivating its transmitter function. Therefore, transporters are significantly expressed in the epidermis. Glutamate uptake is affected by the activities of 2 glutamate transporter subtypes (GLAST and GLT-1) in neuronal cells, such as astrocytes [25, 26]. Thus, we investigated the GLAST and GLT-1 expressions in cultured keratinocytes. While the mRNA expression of GLAST was detectable, that of GLT-1 was nearly undetectable. It has been recognized that, in neuronal cells, astrocytes play an important role in the efficient removal of glutamate from the extracellular space via GLAST [27, 28]. Recently, the glutamate transport function in cultured astrocytes was reported to be regulated mainly by GLAST [10]. It has also been reported that the expressions of glutamate transporters in keratinocytes showed parallels with those in the CNS [5]. Thus, in cultured keratinocytes, the main glutamate transport function might be regulated by GLAST. Furthermore, YKS dose-dependently increased the expression of GLAST mRNA. YKS might enhance the GLAST functions of promoting glutamate transport and excess glutamate uptake. The authors plan to investigate whether YKS will change concentrations of glutamate or not in culture supernatants of keratinocytes which have lost GLAST expression.

Our findings indicate that YKS affects peripheral glutamate signaling in keratinocytes. Glutamine is essential as a transmitter, and dermatitis lesions might produce and release excess glutamate. The investigations indicated that the source of the glutamate was the keratinocytes. This study suggests that, in keratinocytes, YKS controls extracellular glutamate concentrations, suppresses NMDA receptors, and activates glutamate transport. Unfortunately, we could not analyze relation among these findings and mechanisms of each finding. We expect that YKS would inhibit NMDA receptors and activate GLAST by adjusting the extracellular concentration of glutamate in the skin of NC/Nga mice.

Further study is necessary to characterize the mechanism of glutamate signaling and the relationship between the expressions of NMDA and GLAST and excess glutamate in cultured keratinocytes.

Psychological stress affects skin condition in patients with AD [29]. It has been previously demonstrated that psychological stress can trigger AD [29]. We have previously demonstrated that oral administration of YKS alleviates social isolation stress, as indicated by the significantly decreased serum corticosterone levels in NC/Nga mice [20].

In addition, our previous study showed that YKS significantly suppressed scratching and grooming behaviors without exerting a sedative effect on NC/Nga mice [16].

Therefore, these results suggest that YKS affects both the epidermis and the CNS. YKS might be an alternative or a complementary therapeutic option for the treatment of patients with pruritus and dermatitis. Further investigation is anticipated to clarify the precise functional nature of epidermal glutamate signaling and its mediation by YKS.

## References

[1] Fuziwara S, Inoue K, Denda M (2003). NMDA-type glutamate receptor is associated with cutaneous barrier homeostasis. *Journal of Investigative Dermatology*.

[2] Kawakami Z, Kanno H, Ueki T (2009). Neuroprotective effects of yokukansan, a traditional japanese medicine, on glutamate-mediated excitotoxicity in cultured cells. *Neuroscience*.

[3] Wu SZ, Jiang S, Sims TJ, Barger SW (2005). Schwann cells exhibit excitotoxicity consistent with release of NMDA receptor agonists. *Journal of Neuroscience Research*.

[4] Kew JNC, Kemp JA (2005). Ionotropic and metabotropic glutamate receptor structure and pharmacology. *Psychopharmacology*.

[5] Genever PG, Maxfield SJ, Kennovin GD (1999). Evidence for a novel glutamate-mediated signaling pathway in keratinocytes. *Journal of Investigative Dermatology*.

[6] Fischer M, Glanz D, Urbatzka M, Brzoska T, Abels C (2009). Keratinocytes: a source of the transmitter L-glutamate in the epidermis. *Experimental Dermatology*.

[7] Davidson EM, Coggeshall RE, Carlton SM (1997). Peripheral NMDA and non-NMDA glutamate receptors contribute to nociceptive behaviors in the rat formalin test. *NeuroReport*.

[8] Kawakami Z, Ikarashi Y, Kase Y (2010). Glycyrrhizin and its metabolite 18*β*-glycyrrhetinic acid in glycyrrhiza, a constituent herb of yokukansan, ameliorate thiamine deficiency-induced dysfunction of glutamate transport in cultured rat cortical astrocytes. *European Journal of Pharmacology*.

[9] Ikarashi Y, Iizuka S, Imamura S (2009). Effects of yokukansan, a traditional Japanese medicine, on memory disturbance and behavioral and psychological symptoms of dementia in thiamine-deficient rats. *Biological and Pharmaceutical Bulletin*.

[10] Kawakami Z, Ikarashi Y, Kase Y (2011). Isoliquiritigenin is a novel NMDA receptor antagonist in kampo medicine yokukansan.. *Cellular and molecular neurobiology*.

[11] Nahm WK, Philpot BD, Adams MM (2004). Significance of N-methyl-D-aspartate (NMDA) receptor-mediated signaling in human keratinocytes. *Journal of Cellular Physiology*.

[12] Morhenn VB, Murakami M, O’Grady T, Nordberg J, Gallo RL (2004). Characterization of the expression and function of N-methyl-D-aspartate receptor in keratinocytes. *Experimental Dermatology*.

[13] Morhenn VB, Waleh NS, Mansbridge JN (1994). Evidence for an NMDA receptor subunit in human keratinocytes and rat cardiocytes. *European Journal of Pharmacology*.

[14] Hoogduijn MJ, Hitchcock IS, Smit NPM, Gillbro JM, Schallreuter KU, Genever PG (2006). Glutamate receptors on human melanocytes regulate the expression of MiTF. *Pigment Cell Research*.

[15] Fischer M, William T, Helmbold P, Wohlrab J, Marsch WC (2004). Expression of epidermal *N*-methyl-D-aspartate receptors (NMDAR1) depends on formation of the granular layer—analysis in diseases with parakeratotic cornification. *Archives of Dermatological Research*.

[16] Fischer M, Glanz D, William T, Klapperstück T, Wohlrab J, Marsch WCH (2004). N-methyl-D-aspartate receptors influence the intracellular calcium concentration of keratinocytes. *Experimental Dermatology*.

[17] Fischer M, Fiedler E, Seidel C (2006). Cultivated keratinocytes express N-methyl-D-aspartate receptors of the NMDAR2D type. *Archives of Dermatological Research*.

[18] Funakushi N, Yamaguchi T, Jiang J (2011). Ameliorating effect of Yokukansan on the development of atopic dermatitis-like lesions and scratching behavior in socially isolated NC/Nga mice. *Archives of Dermatological Research*.

[19] Suto H, Matsuda H, Mitsuishi K (1999). NC/Nga mice: a mouse model for atopic dermatitis. *International Archives of Allergy and Immunology*.

[20] Jiang J, Yamaguchi T, Funakushi N (2009). Oral administration of Yokukansan inhibits the development of atopic dermatitis-like lesions in isolated NC/Nga mice. *Journal of Dermatological Science*.

[21] Wood JM, Schallreuter KU (2008). A plaidoyer for cutaneous enzymology: our view of some important unanswered questions on the contributions of selected key enzymes to epidermal homeostasis. *Experimental Dermatology*.

[22] Stephenson FA (2001). Subunit characterization of NMDA receptors. *Current Drug Targets*.

[23] Danysz W, Parsons CG (1998). Glycine and N-methyl-D-aspartate receptors: physiological significance and possible therapeutic applications. *Pharmacological Reviews*.

[24] Skerry TM, Genever PG (2001). Glutamate signalling in non-neuronal tissues. *Trends in Pharmacological Sciences*.

[25] Rothstein JD, Martin L, Levey AI (1994). Localization of neuronal and glial glutamate transporters. *Neuron*.

[26] Lehre KP, Levy LM, Ottersen OP, Storm-Mathisen J, Danbolt NC (1995). Differential expression of two glial glutamate transporters in the rat brain: Quantitative and immunocytochemical observations. *Journal of Neuroscience*.

[27] Schlag BD, Vondrasek JR, Munir M (1998). Regulation of the glial Na+-dependent glutamate transporters by cyclic AMP analogs and neurons. *Molecular Pharmacology*.

[28] Kanai Y, Smith CP, Hediger MA (1993). The elusive transporters with a high affinity for glutamate. *Trends in Neurosciences*.

[29] Amano H, Negishi I, Akiyama H, Ishikawa O (2008). Psychological stress can trigger atopic dermatitis in NC/Nga mice: an inhibitory effect of corticotropin-releasing factor. *Neuropsychopharmacology*.

